# From biomedicine to natural history research: EST resources for ambystomatid salamanders

**DOI:** 10.1186/1471-2164-5-54

**Published:** 2004-08-13

**Authors:** Srikrishna Putta, Jeramiah J Smith, John A Walker, Mathieu Rondet, David W Weisrock, James Monaghan, Amy K Samuels, Kevin Kump, David C King, Nicholas J Maness, Bianca Habermann, Elly Tanaka, Susan V Bryant, David M Gardiner, David M Parichy, S Randal Voss

**Affiliations:** 1 Department of Biology, University of Kentucky, Lexington, KY 40506, USA; 2 Department of Developmental and Cell Biology and the Developmental Biology Center, University of California, Irvine, CA 92697, USA; 3 The Life Sciences Consortium, 519 Wartik Laboratory, Penn State University, University Park, PA 16802, USA; 4 Department of Zoology, University of Wisconsin-Madison, 250 N. Mills, Madison, WI 53706, USA; 5 Scionics Computer Innovation GmbH, Pfotenhauerstrasse 110, 01307 Dresden, Germany; 6 Max Planck Institute of Molecular Cell Biology and Genetics, Pfotenhauerstrasse 108, 01307 Dresden, Germany; 7 Section of Integrative Biology and Section of Molecular, Cell and Developmental Biology, Institute for Cellular and Molecular Biology, University of Texas, Austin, TX 78712, USA

## Abstract

**Background:**

Establishing genomic resources for closely related species will provide comparative insights that are crucial for understanding diversity and variability at multiple levels of biological organization. We developed ESTs for Mexican axolotl (*Ambystoma mexicanum*) and Eastern tiger salamander (*A. tigrinum tigrinum*), species with deep and diverse research histories.

**Results:**

Approximately 40,000 quality cDNA sequences were isolated for these species from various tissues, including regenerating limb and tail. These sequences and an existing set of 16,030 cDNA sequences for *A. mexicanum *were processed to yield 35,413 and 20,599 high quality ESTs for *A. mexicanum *and *A. t. tigrinum*, respectively. Because the *A. t. tigrinum *ESTs were obtained primarily from a normalized library, an approximately equal number of contigs were obtained for each species, with 21,091 unique contigs identified overall. The 10,592 contigs that showed significant similarity to sequences from the human RefSeq database reflected a diverse array of molecular functions and biological processes, with many corresponding to genes expressed during spinal cord injury in rat and fin regeneration in zebrafish. To demonstrate the utility of these EST resources, we searched databases to identify probes for regeneration research, characterized intra- and interspecific nucleotide polymorphism, saturated a human – *Ambystoma *synteny group with marker loci, and extended PCR primer sets designed for *A. mexicanum */ *A. t. tigrinum *orthologues to a related tiger salamander species.

**Conclusions:**

Our study highlights the value of developing resources in traditional model systems where the likelihood of information transfer to multiple, closely related taxa is high, thus simultaneously enabling both laboratory and natural history research.

## Background

Establishing genomic resources for closely related species will provide comparative insights that are crucial for understanding diversity and variability at multiple levels of biological organization. Expressed sequence tags (EST) are particularly useful genomic resources because they enable multiple lines of research and can be generated for any organism: ESTs allow the identification of molecular probes for developmental studies, provide clones for DNA microchip construction, reveal candidate genes for mutant phenotypes, and facilitate studies of genome structure and evolution. Furthermore, ESTs provide raw material from which strain-specific polymorphisms can be identified for use in population and quantitative genetic analyses. The utility of such resources can be tailored to target novel characteristics of organisms when ESTs are isolated from cell types and tissues that are actively being used by a particular research community, so as to bias the collection of sequences towards genes of special interest. Finally, EST resources produced for model organisms can greatly facilitate comparative and evolutionary studies when their uses are extended to other, closely related taxa.

Salamanders (urodele amphibians) are traditional model organisms whose popularity was unsurpassed early in the 20^th ^century. At their pinnacle, salamanders were the primary model for early vertebrate development. Embryological studies in particular revealed many basic mechanisms of development, including organizer and inducer regions of developing embryos [1]. Salamanders continue to be important vertebrate model organisms for regeneration because they have by far the greatest capacity to regenerate complex body parts in the adult phase. In contrast to mammals, which are not able to regenerate entire structures or organ systems upon injury or amputation, adult salamanders regenerate their limbs, tail, lens, retina, spinal cord, heart musculature, and jaw [2-7]. In addition, salamanders are the model of choice in a diversity of areas, including vision, embryogenesis, heart development, olfaction, chromosome structure, evolution, ecology, science education, and conservation biology [8-15]. All of these disciplines are in need of genomic resources as fewer than 4100 salamander nucleotide sequences had been deposited in GenBank as of 3/10/04.

Here we describe results from an EST project for two ambystomatid salamanders: the Mexican axolotl, *Ambystoma mexicanum *and the eastern tiger salamander, *A. tigrinum tigrinum*. These two species are members of the Tiger Salamander Complex [16], a group of closely related species and subspecies that are widely distributed in North America. Phylogenetic reconstruction suggests that these species probably arose from a common ancestor about 10–15 million years ago [16]. *Ambystoma mexicanum *has a long research history of over 100 years and is now principally supplied to the research community by the Axolotl Colony [17], while *A. t. tigrinum *is obtained from natural populations in the eastern United States. Although closely related with equally large genomes (32 × 10^9 ^bp)[18], these two species and others of the Complex differ dramatically in life history: *A. mexicanum *is a paedomorphic species that retains many larval features and lives in water throughout it's life cycle while *A. t. tigrinum *undergoes a metamorphosis that is typical of many amphibians. Like many other traditional model organisms of the last century, interest in these two species declined during the rise of genetic models like the fly, zebrafish, and mouse [19]. However, "early" model organisms such as salamanders are beginning to re-attract attention as genome resources can rapidly be developed to exploit the unique features that originally identified their utility for research. We make this point below by showing how the development of ESTs for these two species is enabling research in several areas. Furthermore, we emphasize the value of developing resources in model systems where the likelihood of information transfer to multiple, closely related taxa is high, thus simultaneously enabling both laboratory and natural history research programs.

## Results and Discussion

### Selection of libraries for EST sequencing

Eleven cDNA libraries were constructed using a variety of tissues (Table 1). Pilot sequencing of randomly selected clones revealed that the majority of the non-normalized libraries were moderate to highly redundant for relatively few transcripts. For example, hemoglobin-like transcripts represented 15–25% of the sampled clones from cDNA libraries V1, V2, and V6. Accordingly, we chose to focus our sequencing efforts on the non-normalized MATH library as well as the normalized AG library, which had lower levels of redundancy (5.5 and 0.25% globins, respectively). By concentrating our sequencing efforts on these two libraries we obtained transcripts deriving primarily from regenerating larval tissues in *A. mexicanum *and several non-regenerating larval tissues in *A. t. tigrinum*.

**Table 1:** Tissues selected to make cDNA libraries.

**ID**	**Tissue**	**cDNAs sequenced**
GARD	limb blastema	1029
MATH	limb blastema	16244
V1	tail blastema	1422
V2	brain	3196
V3	liver	792
V4	spleen	337
V5	heart	38
V6	gill	3039
V7	stage 22 embryo	96
AG	liver, gonad, lung, kidney, heart, gill	19871

Further information is found in Methods and Materials.

### EST sequencing and clustering

A total of 46,064 cDNA clones were sequenced, yielding 39,982 high quality sequences for *A. mexicanum *and *A. t. tigrinum *(Table 2). Of these, 3,745 corresponded to mtDNA and were removed from the dataset; complete mtDNA genome data for these and other ambystomatid species will be reported elsewhere. The remaining nuclear ESTs for each species were clustered and assembled separately. We included in our *A. mexicanum *assembly an additional 16,030 high quality ESTs that were generated recently for regenerating tail and neurula stage embryos [20]. Thus, a total of 32,891 and 19,376 ESTs were clustered for *A. mexicanum *and *A. t. tigrinum*, respectively. Using PaCE clustering and CAP3 assembly, a similar number of EST clusters and contigs were identified for each species (Table 2). Overall contig totals were 11,190 and 9,901 for *A. mexicanum *and *A. t. tigrinum *respectively. Thus, although 13,515 more *A. mexicanum *ESTs were assembled, a roughly equivalent number of contigs were obtained for both species. This indicates that EST development was more efficient for *A. t. tigrinum, *presumably because ESTs were obtained primarily from the normalized AG library; indeed, there were approximately twice as many ESTs on average per *A. mexicanum *contig (Table 2). Thus, our EST project yielded an approximately equivalent number of contigs for *A. mexicanum *and *A. t. tigrinum*, and overall we identified > 21,000 different contigs. Assuming that 20% of the contigs correspond to redundant loci, which has been found generally in large EST projects [21], we identified transcripts for approximately 17,000 different ambystomatid loci. If ambystomatid salamanders have approximately the same number of loci as other vertebrates (e.g. [22]), we have isolated roughly half the expected number of genes in the genome.

**Table 2:** EST summary and assembly results.

	***A. mex***	***A. t. tig***
cDNA clones sequenced	21830	24234
high-quality sequences	19383	20599
mt DNA sequence	2522	1223
seqs submitted to NCBI	16861	19376
sequences assembled	32891^a^	19376
PaCE clusters	11381	10226
ESTs in contigs	25457	12676
contigs	3756	3201
singlets	7434	6700
putative transcripts	11190	9901

^a^Includes 16,030 ESTs from [20].

### Identification of vertebrate sequences similar to *Ambystoma *contigs

**Figure 1:**
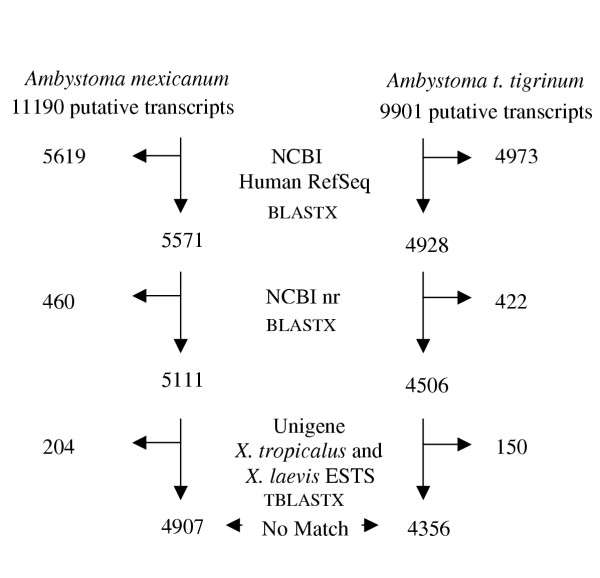
**Results of BLASTX and TBLASTX searches to identify best BLAST hits for *Ambystoma *contigs searched against NCBI human RefSeq, nr, and *Xenopus *Unigene databases**.

We searched all contigs against several vertebrate databases to identify sequences that exhibited significant sequence similarity. As our objective was to reliably annotate as many contigs as possible, we first searched against 19,804 sequences in the NCBI human RefSeq database (Figure 1), which is actively reviewed and curated by biologists. This search revealed 5619 and 4973 "best hit" matches for the *A. mexicanum *and *A. t. tigrinum *EST datasets at a BLASTX threshold of *E *= 10^-7^. The majority of contigs were supported at more stringent E-value thresholds (Table 3). Non-matching contigs were subsequently searched against the Non-Redundant (nr) Protein database and *Xenopus tropicalus *and *X. laevis *UNIGENE ESTs (Figure 1). These later two searches yielded a few hundred more 'best hit' matches, however a relatively large number of ESTs from both ambystomatid species were not similar to any sequences from the databases above. Presumably, these non-matching sequences were obtained from the non-coding regions of transcripts or they contain protein-coding sequences that are novel to salamander. Although the majority are probably of the former type, we did identify 3,273 sequences from the non-matching set that had open reading frames (ORFs) of at least 200 bp, and 911 of these were greater than 300 bp.

**Table 3:** *Ambystoma *contig search of NCBI human RefSeq, nr, and *Xenopus *Unigene databases.

	***A. mex***	***A. t. tig***
# BLASTX Best Matches	6283	5545
< E^-100^	630	870
< E^-50 ^> E^-100^	2015	1990
< E^-20 ^> E^-50^	2153	1595
< E^-10 ^> E^-20^	967	745
< E^-7 ^> E^-10^	518	345

**Figure 2:**
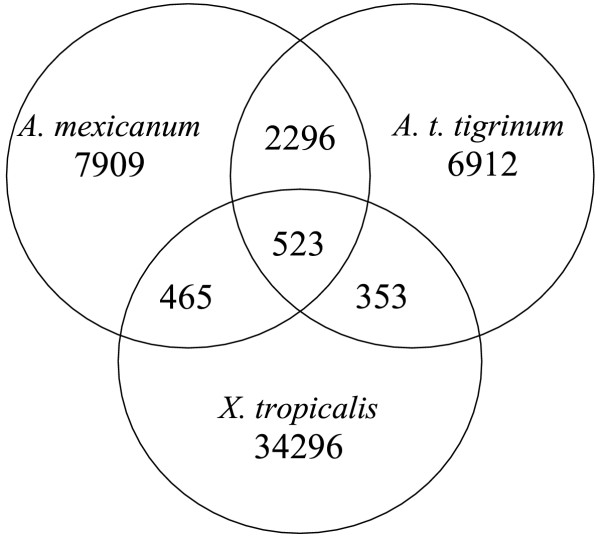
**Venn diagram of BLAST comparisons among amphibian EST projects. **Values provided are numbers of reciprocal best BLAST hits (*E<*10^-20^) among quality masked *A. mexicanum *and *A. t. tigrinum *assemblies and a publicly available *X. tropicalis *EST assembly

The distribution of ESTs among contigs can provide perspective on gene expression when clones are randomly sequenced from non-normalized cDNA libraries. In general, frequently sampled transcripts may be expressed at higher levels. We identified the 20 contigs from *A. mexicanum *and *A. t. tigrinum *that contained the most assembled ESTs (Table 4). The largest *A. t. tigrinum *contigs contained fewer ESTs than the largest *A. mexicanum *contigs, probably because fewer overall *A. t. tigrinum *clones were sequenced, with the majority selected from a normalized library. However, we note that the contig with the most ESTs was identified for *A. t. tigrinum: *delta globin. In both species, transcripts corresponding to globin genes were sampled more frequently than all other loci. This may reflect the fact that amphibians, unlike mammals, have nucleated red blood cells that are transcriptionally active. In addition to globin transcripts, a few other house-keeping genes were identified in common from both species, however the majority of the contigs were unique to each list. Overall, the strategy of sequencing cDNAs from a diverse collection of tissues (from normalized and non-normalized libraries) yielded different sets of highly redundant contigs. Only 25% and 28% of the *A. mexicanum *and *A. t. tigrinum *contigs, respectively, were identified in common (Figure 2). We also note that several hundred contigs were identified in common between Xenopus and *Ambystoma*; this will help facilitate comparative studies among these amphibian models.

**Table 4:** Top 20 contigs with the most assembled ESTs.

**Contig ID**	**# ESTs**	**Best Human Match**	**E-value**
MexCluster_4615_Contig1	415	(NM_000519) delta globin	E^-39^
MexCluster_600_Contig1	354	(NM_182985) ring finger protein 36 isoform a	E^-110^
MexCluster_6279_Contig1	337	(NM_000559) A-gamma globin	E^-32^
MexCluster_10867_Contig1	320	(NM_000558) alpha 1 globin	E^-38^
MexCluster_5357_Contig1	307	(NM_000558) alpha 1 globin	E^-37^
MexCluster_9285_Contig3	285	(NM_001614) actin, gamma 1 propeptide	0
MexCluster_7987_Contig3	252	(NM_001402) eukaryotic translation elongation f1	0
MexCluster_9285_Contig1	240	(NM_001101) beta actin; beta cytoskeletal actin	0
MexCluster_9279_Contig3	218	(NM_000223) keratin 12	E^-113^
MexCluster_11203_Contig1	181	(NM_002032) ferritin, heavy polypeptide 1	E^-70^
MexCluster_8737_Contig2	152	(NM_058242) keratin 6C	E^-131^
MexCluster_3193_Contig1	145	(NM_004499) heterogeneous nuclear ribonucleoprotein	E^-90^
MexCluster_8737_Contig7	134	(NM_058242) keratin 6C	E^-131^
MexCluster_5005_Contig3	132	(NM_031263) heterogeneous nuclear ribonucleoprotein	E^-124^
MexCluster_6225_Contig1	125	(NM_001152) solute carrier family 25, member 5	E^-151^
MexCluster_1066_Contig1	122	[31015660] IMAGE:6953586	E^-16^
MexCluster_8737_Contig4	114	(NM_058242) keratin 6C; keratin, epidermal type II	E^-132^
MexCluster_8187_Contig2	113	(NM_005507) cofilin 1 (non-muscle)	E^-65^
MexCluster_2761_Contig1	109	(NM_001961) eukaryotic translation elongation factor2	0
MexCluster_9187_Contig1	105	(NM_007355) heat shock 90 kDa protein 1, beta	0
***A. t. tigrinum***			
TigCluster_6298_Contig1	654	(NM_000519) delta globin	E^-38^
TigCluster_10099_Contig2	193	(NM_001614) actin, gamma 1 propeptide	0
TigCluster_6470_Contig1	167	(NM_000558) alpha 1 globin	E^-39^
TigCluster_9728_Contig2	142	(NM_000477) albumin precursor	E^-140^
TigCluster_6594_Contig1	117	(NM_001402) eukaryotic translation elongation f1	0
TigCluster_5960_Contig1	91	(NM_001101) beta actin; beta cytoskeletal actin	0
TigCluster_7383_Contig1	77	(NM_001614) actin, gamma 1 propeptide	0
TigCluster_6645_Contig1	76	(NM_001063) transferrin	0
TigCluster_7226_Contig4	74	(NM_006009) tubulin, alpha 3	E^-160^
TigCluster_7191_Contig1	67	(NM_019016) keratin 24	E^-89^
TigCluster_10121_Contig1	64	(NM_005141) fibrinogen, beta chain preproprotein	0
TigCluster_6705_Contig1	63	(NM_000558) alpha 1 globin	E^-39^
TigCluster_7854_Contig1	62	(NM_021870) fibrinogen, gamma chain isoform	E^-121^
TigCluster_6139_Contig1	52	(NM_001404) eukaryotic translation elongation f1	0
TigCluster_7226_Contig2	51	(NM_006009) tubulin, alpha 3	0
TigCluster_10231_Contig1	44	(NM_003018) surfactant, pulmonary-associated prot.	E^-08^
TigCluster_6619_Contig1	36	(NM_000041) apolipoprotein E	E^-38^
TigCluster_7232_Contig2	35	(NM_003651) cold shock domain protein A	E^-46^
TigCluster_5768_Contig1	34	(NM_003380) vimentin	E^-177^
TigCluster_9784_Contig3	32	|XP_218445.1| similar to RIKEN cDNA 1810065E05	E^-15^

### Functional annotation

For the 10,592 contigs that showed significant similarity to sequences from the human RefSeq database, we obtained Gene Ontology (23) information to describe ESTs in functional terms. Although there are hundreds of possible annotations, we chose a list of descriptors for molecular and biological processes that we believe are of interest for research programs currently utilizing salamanders as model organisms (Table 5). In all searches, we counted each match between a contig and a RefSeq sequence as identifying a different ambystomatid gene, even when different contigs matched the same RefSeq reference. In almost all cases, approximately the same number of matches was found per functional descriptor for both species. This was not simply because the same loci were being identified for both species, as only 20% of the total number of searched contigs shared sufficient identity (BLASTN; *E<*10^-80 ^or *E<*10^-20^) to be potential homologues. In this sense, the sequencing effort between these two species was complementary in yielding a more diverse collection of ESTs that were highly similar to human gene sequences.

**Table 5:** Functional annotation of contigs

	***A. mex***	***A. t. tig***
**Molecular Function (0016209)**		
antioxidant (0016209)	25	29
binding (0005488)	3117	2578
chaparone (0003754)	100	85
enzyme regulation (003023)	193	223
motor (0003774)	73	75
signal transduction (0004871)	344	375
structural protein (0005198)	501	411
transcriptional reg. (0030528)	296	221
translational reg. (0045182)	94	59
bone remodeling (0046849)	8	8
circulation (0008015)	23	78
immune response (000695)	182	263
respiratory ex. (0009605)	254	288
respiratory in. (0009719)	72	58
stress (0006950)	263	320
**Biological Process (0008150)**		
Cellular (0009987)		
activation (0001775)	4	6
aging and death (0008219)	158	148
communication (0007154)	701	696
differentiation (0030154)	31	20
extracellular mat. (0043062)	4	4
growth and main. (0008151)	1731	1445
migration (0016477)	8	14
motility (0006928)	163	154
Developmental (0007275)		
aging (0007568)	32	21
embryonic (0009790)	6	1
growth (0040007)	2	2
morphogenesis (0009653)	350	272
pigment (0048066)	13	26
post embryonic (0009791)	8	13
reproduction (0000003)	42	27
Physiological (0007582)		
coagulation (0050817)	22	73
death and aging (0016265)	159	148
homeostasis (0042592)	22	27
metabolism (0008152)	3059	2513
secretion (0046903)	9	16
sex differentiation (0007548)	3	2

Numbers in parentheses reference GO numbers [23].

### Informatic searches for regeneration probes

The value of a salamander model to regeneration research will ultimately rest on the ease in which data and results can be cross-referenced to other vertebrate models. For example, differences in the ability of mammals and salamanders to regenerate spinal cord may reflect differences in the way cells of the ependymal layer respond to injury. As is observed in salamanders, ependymal cells in adult mammals also proliferate and differentiate after spinal cord injury (SCI) [24,25]; immediately after contusion injury in adult rat, ependymal cell numbers increase and proliferation continues for at least 4 days [[26]; but see [27]]. Rat ependymal cells share some of the same gene expression and protein properties of embryonic stem cells [28], however no new neurons have been observed to derive from these cells *in vivo *after SCI [29]. Thus, although endogenous neural progenitors of the ependymal layer may have latent regenerative potential in adult mammals, this potential is not realized. Several recently completed microarray analyses of spinal cord injury in rat now make it possible to cross-reference information between amphibians and mammals. For example, we searched the complete list of significantly up and down regulated genes from Carmel et al. [30] and Song et al. [31] against all *Ambystoma *ESTs. Based upon amino acid sequence similarity of translated ESTs (TBLASTX; *E*<10^-7^), we identified DNA sequences corresponding to 69 of these 164 SCI rat genes (Table 6). It is likely that we have sequence corresponding to other presumptive orthologues from this list as many of our ESTs only contain a portion of the coding sequence or the untranslated regions (UTR), and in many cases our searches identified closely related gene family members. Thus, many of the genes that show interesting expression patterns after SCI in rat can now be examined in salamander.

**Table 6:** *Ambystoma *contigs that show sequence similarity to rat spinal cord injury genes.

**Ambystoma Contig ID**	**RAT cDNA clone**	**E-value**
MexCluster_7440_Contig1	gi|1150557|c-myc, exon 2	E^-29^
MexCluster_4624_Contig1	gi|1468968| brain acyl-CoA synthtase II	E^-09^
TigCluster_4083_Contig1		E^-09^
TigSingletonClusters_Salamander_4_G20_ab1	gi|1552375| SKR6 gene, a CB1 cannabinoid recept.	E^-08^
MexSingletonClusters_NT009B_B04	gi|17352488| cyclin ania-6a	E^-46^
TigCluster_3719_Contig1		E^-114^
TigCluster_8423_Contig1	gi|1778068| binding zyginI	E^-102^
TigCluster_7064_Contig1	gi|1836160| Ca2+/calmodulin-dependent	E^-20^
MexCluster_3225_Contig1	gi|1906612| Rattus norvegicus CXC chemokine	E^-68^
TigSingletonClusters_Salamander_13_F03_ab1		E^-38^
MexSingletonClusters_BL285B_A06	gi|203042| (Na+, K+)-ATPase-beta-2 subunit	E^-63^
TigCluster_6994_Contig1		E^-65^
MexSingletonClusters_BL014B_F12	gi|203048| plasma membrane Ca2+ ATPase-isoform 2	E^-112^
TigSingletonClusters_Salamander_5_F07_ab1		E^-92^
MexCluster_1251_Contig1	gi|203167| GTP-binding protein (G-alpha-i1)	E^-110^
TigSingletonClusters_Salamander_3_P14_ab1		E^-152^
TigSingletonClusters_Salamander_22_B01_ab1	gi|203336| catechol-O-methyltransferase	E^-47^
TigSingletonClusters_Salamander_17_N04_ab1	gi|203467| voltage-gated K+ channel protein (RK5)	E^-08^
MexSingletonClusters_v1_p8_c16_triplex5ld_	gi|203583| cytosolic retinol-binding protein (CRBP)	E^-77^
TigCluster_6321_Contig1		E^-18^
MexCluster_5399_Contig1	gi|204647| heme oxygenase gene	E^-67^
TigCluster_2577_Contig1		E^-67^
MexCluster_4647_Contig1	gi|204664| heat shock protein 27 (Hsp27)	E^-83^
TigSingletonClusters_Salamander_12_M05_ab1		E^-51^
MexSingletonClusters_BL285C_F02	gi|205404| metabotropic glutamate receptor 3	E^-41^
TigSingletonClusters_Salamander_2_B24_ab1	gi|205508| myelin/oligodendrocyte glycoprotein	E^-26^
TigCluster_5740_V2_p10_M20_TriplEx5ld_	gi|205531| metallothionein-2 and metallothionein 1	E^-08^
TigSingletonClusters_V2_p5_A2_TriplEx5ld_	gi|205537| microtubule-associated protein 1A	E^-59^
MexCluster_1645_Contig1	gi|205633| Na, K-ATPase alpha-2 subunit	E^-149^
TigSingletonClusters_Contig328		0
TigSingletonClusters_Contig45	gi|205683| smallest neurofilament protein (NF-L)	E^-63^
MexSingletonClusters_NT016A_A09	gi|205693| nerve growth factor-induced (NGFI-A)	E^-95^
TigSingletonClusters_I09_Ag2_p9_K24_M13R		E^-24^
MexSingletonClusters_NT007A_E07	gi|205754| neuronal protein (NP25)	E^-64^
TigCluster_7148_Contig1		E^-57^
MexCluster_9504_Contig1	gi|206161| peripheral-type benzodiazepine receptor	E^-73^
MexSingletonClusters_BL016B_B02	gi|206166| protein kinase C type III	E^-36^
TigCluster_981_Contig1		E^-27^
MexSingletonClusters_nm_19_k3_t3_	gi|206170| brain type II Ca2+/calmodulin-dependent	E^-117^
MexSingletonClusters_v11_p42_j20_t3_049_ab1 gi|207138| norvegicus syntaxin B		1e^-079^
MexSingletonClusters_nm_14_h19_t3_	gi|207473| neural receptor protein-tyrosine kinase	E^-40^
TigSingletonClusters_Contig336		E^-34^
TigSingletonClusters_E10_Ag2_p18_O19_M13	gi|2116627| SNAP-25A	E^-123^
MexCluster_211_Contig1	gi|220713| calcineurin A alpha	E^-63^
TigSingletonClusters_Salamander_7_K14_ab1		E^-87^
MexSingletonClusters_NT014A_G03	gi|220839| platelet-derived growth factor A chain	E^-21^
TigSingletonClusters_Salamander_9_M15_ab1		E^-56^
TigSingletonClusters_Salamander_19_M06_ab1	gi|2501807| brain digoxin carrier protein	E^-55^
MexSingletonClusters_Contig100	gi|2746069| MAP-kinase phosphatase (cpg21)	E^-108^
TigSingletonClusters_Salamander_11_A16_ab1		E^-70^
MexCluster_8345_Contig1	gi|2832312| survival motor neuron (smn)	E^-40^
TigCluster_8032_Contig1		E^-49^
MexCluster_3580_Contig1	gi|294567| heat shock protein 70 (HSP70)	0
TigCluster_8592_Contig2		E^-161^
TigSingletonClusters_Salamander_17_N08_ab1	gi|2961528| carboxyl-terminal PDZ	E^-10^
MexSingletonClusters_BL286C_D09	gi|298325| sodium-dependent neurotransmitter tran.	E^-12^
TigSingletonClusters_Contig95		E^-22^
MexSingletonClusters_Contig461	gi|2996031| brain finger protein (BFP)	E^-08^
TigSingletonClusters_Salamander_11_O19_ab1		E^-23^
TigSingletonClusters_E16_Ag2_p8_O20_M13R	gi|3135196| Ca2+/calmodulin-dependent	E^-33^
MexSingletonClusters_Contig188	gi|3252500| CC chemokine receptor protein	E^-15^
MexCluster_6961_Contig1	gi|3319323| suppressor of cytokine signaling-3	E^-08^
MexSingletonClusters_nm_14_p15_t3_	gi|349552| P-selectin	E^-16^
TigCluster_218_Contig2		E^-99^
MexSingletonClusters_Contig506	gi|3707306| Normalized rat embryo, cDNA clone	E^-14^
TigSingletonClusters_I16_Ag2_p5_N7_M13R	gi|3711670| Normalized rat muscle, cDNA clone	E^-35^
MexSingletonClusters_V1_p1_a10_Triplex5Ld	gi|3727094| Normalized rat ovary, cDNA clone	E^-15^
TigSingletonClusters_v2_p1_D20_triplex5ld		E^-16^
MexSingletonClusters_NT005B_F02	gi|3811504| Normalized rat brain, cDNA clone	E^-35^
TigSingletonClusters_Salamander_22_I04_ab1		E^-34^
TigSingletonClusters_Ag2_p34_N23_M13R	gi|405556| adenylyl cyclase-activated serotonin	E^-17^
TigSingletonClusters_Salamander_1_H02_ab1	gi|4103371| putative potassium channel TWIK	E^-22^
MexCluster_4589_Contig1	gi|4135567| Normalized rat embryo, cDNA clone	E^-32^
TigSingletonClusters_Contig220		E^-09^
TigCluster_4093_Contig1	gi|4228395| cDNA clone UI-R-A0-bc-h-02-0-UI	E^-104^
MexSingletonClusters_nm_21_2_m7_t3_	gi|425471| nuclear factor kappa B p105 subunit	E^-22^
TigCluster_8535_Contig1		E^-11^
MexSingletonClusters_v6_p1_j6_triplex5_1ld_	gi|430718| Sprague Dawley inducible nitric oxide	E^-13^
TigSingletonClusters_Salamander_15_D22_ab1		E^-41^
MexCluster_3498_Contig1	gi|436934| Sprague Dawley protein kinase C rec.	0
TigCluster_6648_Contig1		0
MexSingletonClusters_BL279A_B12	gi|464196| phosphodiesterase I	E^-49^
TigSingletonClusters_Salamander_25_P03_ab1		E^-75^
MexCluster_8708_Contig1	gi|466438| 40kDa ribosomal protein	E^-168^
TigCluster_5877_Contig1		E^-168^
MexSingletonClusters_nm_14_a9_t3_	gi|493208| stress activated protein kinase alpha II	E^-51^
TigSingletonClusters_Salamander_11_A13_ab1	gi|517393| tau microtubule-associated protein	E^-44^
TigSingletonClusters_Salamander_12_J14_ab1	gi|55933| c-fos	E^-26^
MexSingletonClusters_nm_21_2_l13_t3_	gi|56822| major synaptic vesicel protein p38	E^-39^
TigCluster_2065_Contig1		E^-50^
MexCluster_10965_Contig1	gi|56828| nuclear oncoprotein p53	E^-75^
TigCluster_5315_Contig1		E^-66^
MexCluster_4245_Contig1	gi|56909| pJunB gene	E^-50^
TigSingletonClusters_G05_Ag2_p9_G8_M13R		E^-09^
MexSingletonClusters_NT013D_C12	gi|56919| region fragment for protein kinase C	E^-33^
TigSingletonClusters_Salamander_21_H19_ab1		E^-24^
MexCluster_9585_Contig1	gi|57007| ras-related mRNA rab3	E^-61^
TigCluster_4885_Contig1		E^-63^
TigSingletonClusters_Salamander_1_M03_ab1	gi|57238| silencer factor B	E^-13^
MexSingletonClusters_NT008B_D05	gi|57341| transforming growth factor-beta 1	E^-13^
TigSingletonClusters_Salamander_24_I16_ab1		E^-20^
MexCluster_9533_Contig1	gi|57479| vimentin	0
TigCluster_5768_Contig1		0
MexSingletonClusters_BL283B_A11	gi|596053| immediate early gene transcription	E^-12^
TigSingletonClusters_Salamander_13_J19_ab1		E^-16^
MexSingletonClusters_v6_p4_j2_triplex5_1ld_	gi|790632| macrophage inflammatory protein-1alpha	E^-22^
TigCluster_2146_Contig1	gi|951175| limbic system-associated membrane prot.	E^-11^
MexSingletonClusters_v11_p54_o4_t3_	gi|971274| neurodegeneration associated protein 1	E^-09^
TigSingletonClusters_Salamander_2_J12_ab1		E^-11^

**Figure 3:**
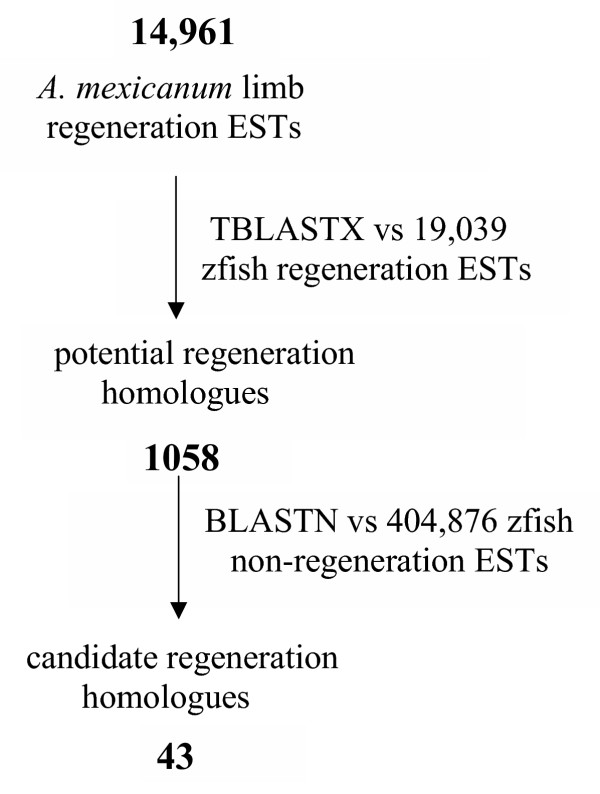
**Results of BLASTN and TBLASTX searches to identify best BLAST hits for *A. mexicanum *regeneration ESTs searched against zebrafish EST databases. **A total of 14,961 *A. mexicanum *limb regeneration ESTs were assembled into 4485 contigs for this search.

Similar gene expression programs may underlie regeneration of vertebrate appendages such as fish fins and tetrapod limbs. Regeneration could depend on reiterative expression of genes that function in patterning, morphogenesis, and metabolism during normal development and homeostasis. Or, regeneration could depend in part on novel genes that function exclusively in this process. We investigated these alternatives by searching *A. mexicanum *limb regeneration ESTs against UNIGENE zebrafish fin regeneration ESTs (Figure 3). This search identified 1357 significant BLAST hits (TBLASTX; *E*<10^-7^) that corresponded to 1058 unique zebrafish ESTs. We then asked whether any of these potential regeneration homologues were represented uniquely in limb and fin regeneration databases (and not in databases derived from other zebrafish tissues). A search of the 1058 zebrafish ESTs against > 400,000 zebrafish ESTs that were sampled from non-regenerating tissues revealed 43 that were unique to the zebrafish regeneration database (Table 7). Conceivably, these 43 ESTs may represent transcripts important to appendage regeneration. For example, our search identified several genes (e.g. *hspc128*, pre-*B-cell colony enhancing factor 1*, *galectin 4*, *galectin 8*) that may be expressed in progenitor cells that proliferate and differentiate during appendage regeneration. Overall, our results suggest that regeneration is achieved largely through the reiterative expression of genes having additional functions in other developmental contexts, however a small number of genes may be expressed uniquely during appendage regeneration.

**Table 7:** *Ambystoma *limb regeneration contigs that show sequence similarity to zebrafish fin regeneration ESTs

**Mex. Contigs**	**Human ID**	**E-value**	**Zfish ID**	**E-value**
Contig94	gi|10835079|	1e^-63^	gnl|UG|Dr#S12319632	1e^-58^
nm_30_a11_t3_	gi|32306539|	1e^-58^	gnl|UG|Dr#S12312602	1e^-35^
Contig615	gi|4502693|	1e^-70^	gnl|UG|Dr#S12313407	1e^-34^
nm_23_l13_t3_	No Human Hit		gnl|UG|Dr#S12320916	1e^-31^
nm_9_e22_t3_	gi|4758788|	1e^-98^	gnl|UG|Dr#S12309914	1e^-29^
nm_8_l17_t3_	gi|21361310|	1e^-16^	gnl|UG|Dr#S12313396	1e^-27^
Contig531	gi|13775198|	1e^-27^	gnl|UG|Dr#S12309680	1e^-26^
Contig152	gi|5453712|	1e^-32^	gnl|UG|Dr#S12239884	1e^-26^
nm_32h_j20_t3_	gi|39777601|	1e^-79^	gnl|UG|Dr#S12136499	1e^-25^
Contig1011	gi|39752675|	1e^-65^	gnl|UG|Dr#S12136499	1e^-24^
v11_p50_b24_t3_	gi|41208832|	1e^-36^	gnl|UG|Dr#S12319219	1e^-23^
Contig589	gi|4506505|	1e^-56^	gnl|UG|Dr#S12312662	1e^-22^
Contig785	gi|33695095|	1e^-61^	gnl|UG|Dr#S12264765	1e^-22^
Contig157	gi|21361122|	1e^-138^	gnl|UG|Dr#S12313094	1e^-21^
v11_p42_j20_t3_049_ab1	gi|47591841|	1e^-100^	gnl|UG|Dr#S12137806	1e^-21^
Contig610	gi|10801345|	1e^-114^	gnl|UG|Dr#S12310326	1e^-20^
nm_27_o1_t3_	gi|7706429|	1e^-72^	gnl|UG|Dr#S12310422	1e^-19^
Contig439	gi|4504799|	1e^-25^	gnl|UG|Dr#S12309233	1e^-19^
nm_31_d5_t3_	gi|8923956|	1e^-50^	gnl|UG|Dr#S12264745	1e^-17^
v11_p41_h12_t3_026_ab1	No Human Hit		gnl|UG|Dr#S12320916	1e^-17^
Contig129	gi|34932414|	1e^-103^	gnl|UG|Dr#S12313534	1e^-17^
nm_14_j21_t3_	gi|4505325|	1e^-42^	gnl|UG|Dr#S12136571	1e^-17^
Contig1321	gi|4501857|	1e^-80^	gnl|UG|Dr#S12309233	1e^-17^
nm_19_k3_t3_	gi|26051212|	1e^-106^	gnl|UG|Dr#S12137637	1e^-17^
Contig488	gi|4557525|	1e^-105^	gnl|UG|Dr#S12311975	1e^-15^
nm_35h_k19_t3_	gi|16950607|	1e^-43^	gnl|UG|Dr#S12196214	1e^-15^
Contig195	gi|4557231|	1e^-99^	gnl|UG|Dr#S12309233	1e^-14^
nm_14_h19_t3_	gi|4503787|	1e^-86^	gnl|UG|Dr#S12310912	1e^-13^
v11_p51_d20_t3_	gi|30520322|	1e^-19^	gnl|UG|Dr#S12321150	1e^-13^
g3-n14	gi|13654278|	1e^-23^	gnl|UG|Dr#S12318856	1e^-13^
nm_29_f2_t3_	gi|4506517|	1e^-65^	gnl|UG|Dr#S12312662	1e^-13^
g4-h23	gi|24111250|	1e^-33^	gnl|UG|Dr#S12312651	1e^-13^
Math_p2_A2_T3_	No human Hit		gnl|UG|Dr#S12078998	1e^-13^
nm_35h_f4_t3_	gi|41148476|	1e^-67^	gnl|UG|Dr#S12319663	1e^-13^
Contig952	gi|21264558|	1e^-61^	gnl|UG|Dr#S12318843	1e^-12^
g4-g21	gi|11995474|	1e^-65^	gnl|UG|Dr#S12192716	1e^-12^
Contig854	gi|8922789|	1e^-117^	gnl|UG|Dr#S12313534	1e^-11^
Contig1105	gi|6912638||	1e^-83^	gnl|UG|Dr#S12079967	1e^-11^
nm_26_f7_t3_	gi|30181238|	1e^-83^	gnl|UG|Dr#S12319880	1e^-11^
Contig949	gi|21284385|	1e^-68^	gnl|UG|Dr#S12290856	1e^-11^
g3-n3	gi|18490991|	1e^-64^	gnl|UG|Dr#S12320832	1e^-10^
v11_p41_m16_t3_007_ab1	gi|4885661|	1e^-33^	gnl|UG|Dr#S12310912	1e^-10^
Contig653	gi|4505047|	1e^-124^	gnl|UG|Dr#S12239868	1e^-09^
Contig1349	gi|9665259|	1e^-46^	gnl|UG|Dr#S12320840	1e^-09^
6h12	gi|31317231|	1e^-43^	gnl|UG|Dr#S12321311	1e^-09^
v11_p43h_i14_t3_070_ab1	No Human Hit		gnl|UG|Dr#S12320916	1e^-09^
nm_35h_d11_t3_	gi|7661790|	1e^-35^	gnl|UG|Dr#S12196146	1e^-09^
nm_35h_k22_t3_	gi|5031977|	1e^-124^	gnl|UG|Dr#S12242267	1e^-09^
v11_p48_g2_t3_087_ab1	gi|11496277|	1e^-60^	gnl|UG|Dr#S12312396	1e^-09^
nm_30_e11_t3_	gi|32483357|	1e^-56^	gnl|UG|Dr#S12309103	1e^-08^
nm_28_f23_t3_	gi|42544191|	1e^-25^	gnl|UG|Dr#S12239884	1e^-08^
nm_12_p16_t3_	gi|21361553|	1e^-21^	gnl|UG|Dr#S12310912	1e^-08^
nm_32h_a8_t3_	gi|11386179|	1e^-22^	gnl|UG|Dr#S12312152	1e^-08^

Human RefSeq sequence ID's are provided to allow cross-referencing.

### DNA sequence polymorphisms within and between *A. mexicanum *and *A. t. tigrinum*

The identification of single nucleotide polymorphisms (SNPs) within and between orthologous sequences of *A. mexicanum *and *A. t. tigrinum *is needed to develop DNA markers for genome mapping [32], quantitative genetic analysis [33], and population genetics [34]. We estimated within species polymorphism for both species by calculating the frequency of SNPs among ESTs within the 20 largest contigs (Table 4). These analyses considered a total of 30,638 base positions for *A. mexicanum *and 18,765 base positions for *A. t. tigrinum*. Two classes of polymorphism were considered in this analysis: those occurring at moderate (identified in 10–30% of the EST sequences) and high frequencies (identified in at least 30% of the EST sequences). Within the *A. mexicanum *contigs, 0.49% and 0.06% of positions were polymorphic at moderate and high frequency, while higher levels of polymorphism were observed for *A. t. tigrinum *(1.41% and 0.20%). Higher levels of polymorphism are expected for *A. t. tigrinum *because they exist in larger, out-bred populations in nature.

To identify SNPs between species, we had to first identify presumptive, interspecific orthologues. We did this by performing BLASTN searches between the *A. mexicanum *and *A. t. tigrinum *assemblies, and the resulting alignments were filtered to retain only those alignments between sequences that were one another's reciprocal best BLAST hit. As expected, the number of reciprocal 'best hits' varied depending upon the *E *value threshold, although increasing the *E *threshold by several orders of magnitude had a disproportionately small effect on the overall total length of BLAST alignments. A threshold of *E<*10^-80^yielded 2414 alignments encompassing a total of 1.25 Mbp from each species, whereas a threshold of *E<*10^-20 ^yielded 2820 alignments encompassing a total of 1.32 Mbp. The percent sequence identity of alignments was very high among presumptive orthologues, ranging from 84–100% at the more stringent *E *threshold of *E<*10^-80^. On average, *A. mexicanum *and *A. t. tigrinum *transcripts are estimated to be 97% identical at the nucleotide level, including both protein coding and UTR sequence. This estimate for nuclear sequence identity is surprisingly similar to estimates obtained from complete mtDNA reference sequences for these species (96%, unpublished data), and to estimates for partial mtDNA sequence data obtained from multiple natural populations [16]. These results are consistent with the idea that mitochondrial mutation rates are lower in cold versus warm-blooded vertebrates [35]. From a resource perspective, the high level of sequence identity observed between these species suggests that informatics will enable rapidly the development of probes between these and other species of the *A. tigrinum *complex.

### Extending EST resources to other ambystomatid species

Relatively little DNA sequence has been obtained from species that are closely related to commonly used model organisms, and yet, such extensions would greatly facilitate genetic studies of natural phenotypes, population structures, species boundaries, and conservatism and divergence of developmental mechanisms. Like many amphibian species that are threatened by extinction, many of these ambystomatid salamanders are currently in need of population genetic studies to inform conservation and management strategies [e.g. [13]]. We characterized SNPs from orthologous *A. mexicanum *and *A. t. tigrinum *ESTs and extended this information to develop informative molecular markers for a related species, *A. ordinarium*. *Ambystoma ordinarium *is a stream dwelling paedomorph endemic to high elevation habitats in central Mexico [36]. This species is particularly interesting from an ecological and evolutionary standpoint because it harbors a high level of intraspecific mitochondrial variation, and as an independently derived stream paedomorph, is unique among the typically pond-breeding tiger salamanders. As a reference of molecular divergence, *Ambystoma ordinarium *shares approximately 98 and 97% mtDNA sequence identity with *A. mexicanum *and *A. t. tigrinum *respectively [16].

To identify informative markers for *A. ordinarium*, *A. mexicanum and A. t. tigrinum *EST contigs were aligned to identify orthologous genes with species-specific sequence variations (SNPs or Insertion/Deletions = INDELs). Primer pairs corresponding to 123 ESTs (Table 8) were screened by PCR using a pool of DNA template made from individuals of 10 *A. ordinarium *populations. Seventy-nine percent (N = 97) of the primer pairs yielded amplification products that were approximately the same size as corresponding *A. mexicanum *and *A. t. tigrinum *fragments, using only a single set of PCR conditions. To estimate the frequency of intraspecific DNA sequence polymorphism among this set of DNA marker loci, 43 loci were sequenced using a single individual sampled randomly from each of the 10 populations, which span the geographic range of *A. ordinarium*. At least one polymorphic site was observed for 20 of the sequenced loci, with the frequency of polymorphisms dependent upon the size of the DNA fragment amplified. Our results suggest that the vast majority of primer sets designed for *A. mexicanum */ *A. t. tigrinum *EST orthologues can be used to amplify the corresponding sequence in a related *A. tigrinum *complex species, and for small DNA fragments in the range of 150–500 bp, approximately half are expected to have informative polymorphisms.

**Table 8:** EST loci used in a population-level PCR amplification screen in *A. ordinarium*

**Locus ID**	**Forward Primer 5' to 3'**	**Reverse Primer 5' to 3'**
1F8	AAGAAGGTCGGGATTGTGGGTAA	CAGCCTTCCTCTTCATCTTTGTCTTG
1H3	GGCAAATGCTGGTCCCAACACAAA	GGACAACACTGCCAAATACCACAT
2C8	GCAAGCACCAGCCACATAAAG	GGCCACCATAACCACTCTGCT
3B10	TCAAAACGAATAAGGGAAGAGCGACTG	TTGCCCCCATAATAAGCCATCCATC
5E7	ACGCTTCGCTGGGGTTGACAT	CGGTAGGATTTCTGGTAGCGAGCAC
5F4	CCGAGATGAGATTTATAGAAGGAC	TAGGGGAAGTTAAACATAGATAGAA
6A3	GTTTATGAAGGCGAGAGGGCTATGACCA	ATCTTGTTCTCCTCGCCAGTGCTCTTGT
6B1	TGATGCTGGCGAGTACAAACCCCCTTCT	TTTACCATTCCTTCCCTTCGGCAGCACA
6B3	ACCACGTGCTGTCTTCCCATCCAT	ACGAAGCTCATTGTAGAAGGTGTG
6B4	CCCACGATGAATTGGAATTGGACAT	CTGCCTGCCAGACCTACAGACTATCGT
6C4	ATGGCGCCAAAGTGATGAGTA	GGGCCAGGCACACGACCACAAT
6D2	ATCAAGGCTGGCATGGTGGTCA	GGGGGTCGTTCTTGCTGTCA
6H8	GAAGAAGACAGAAACGCAGGAGAAAAAC	CGGGCGGGGGCGGGTCACAGTAAAAC
BL005B_A01.5.1	GACAGGTCATGAACTTTTGAAAATAA	AAAGTATATGTACCAAATGGGAGAGC
BL006A_G07.5.1	GATGTCCTCTCCACTATACAAGTGTG	GTTTGACTTGTCACCACTTTATCAAC
BL012D_F02.5.1	ACAGCCAGAAATAGAAACTTTGAACT	TGAAAGTATGTATTGTTTTCACAGGG
BL013C_E01.5.1	AGGATGAAATAATATGCTGTGCTTC	ACCGTGATAAACTCCATCCCTT
BL014D_B11.5.1	AGCAAAACTCCTCTATGAATCTCG	ATTGCACACTAAATAGGTGAATACGA
BL279A_G10.5.1	ATGGCAGGATGAAGAAAGACAT	ATGCACTTTGGACCCACTGAG
Et.fasta.Contig1023.5.1	TGTGGTTATTGGACTACTTCACTCTC	AAACGTCCATTTGACACTGTATTTTA
Et.fasta.Contig1166.5.1	GAATGAAGAGAAAATGTTTTGAAGGT	GCACAGTATTGGCTATGAGCAC
Et.fasta.Contig1311.5.1	AGAAAACTGTGTCAAGCTTATTTTCC	CAACTTAGTGTTCACATTTCTGAGGT
Et.fasta.Contig1335.5.1	CCACTTATGGTAGTTCCCACTTTTAT	GCTAAAGAATACCAAGAACCTTTGAC
Et.fasta.Contig1381.5.1	GTCACAGGTATAACATTGAAAGGATG	TAAATGAATCAAACATTGAAGAGAGC
Et.fasta.Contig1459.5.1	ATAACAAGGACATGTTCTGCTGG	CTAGCAGAACCCTGTATAGCCTG
Et.fasta.Contig1506.5.1	AGGATATCCGCTCAGAAATATGAAG	CTGACCACTTGCAAAACTTACTACCT
Et.fasta.Contig1578.5.1	CCTAGAACATTACCAAAACAGACTCA	AATGAAGAAGTATTGCATGTGAGAAC
Et.fasta.Contig1647.5.1	GTACAACGTCAGGCAAAGCTATTCT	ATCTCCAACACCGTGGCTAAT
Et.fasta.Contig1717.5.1	GAACTTGTTGGCAGGTTTCTCTT	CTAGTGATAGGTTGGACATACCAGAG
Et.fasta.Contig1796.5.1	TGTGGGTATGTATATGGCTAACTTGT	AGATTTTATGTGCTACTGCATTTACG
Et.fasta.Contig1908.5.1	CTCATGACTTAATTGCTGTTCTTCG	ATAACCATTCTGAGGTTTTGAGTTG
Et.fasta.Contig1941.5.1	ATCTCCTGCTTCATCTCTTGATTTAT	TAACAGATTTAATAAACGTCCCCTTC
Et.fasta.Contig1943.5.1	AGTACGATGAATCTGGTCCTTCAAT	CCACAATACTGACATACTCTGGTCTT
Et.fasta.Contig325.5.1	GTGAAGTCAGTGAGTAAAGTCCATGT	CTAGGATACCAGTGGGAGAGTGTAAT
Et.fasta.Contig330.5.1	GTCATCACCTCCACTACTTCACAAG	TTTTGGCACTGTAAGATTCTATGAAC
Et.fasta.Contig536.5.1	CCTTAGGTAGAACAGACTGAAGCAG	GAAACATGAAACTGGACTTGTTTTAG
Et.fasta.Contig917.5.1	GGATGCAGATTCTTCCTATTTTACTC	CTGGTCACTTTACTTGTTTTCAGTGT
Et.fasta.Contig926.5.1	TTCATCACATTCTACTTCACAAATCA	CTAGGCAAGCAAGCTTTCTAATAGTT
Et.fasta.Contig93.5.1	GAATAAAAGCAACAATTGCAGAGTTA	CTCGACTCCTTCTACGATCTCTACTC
Et.fasta.Contig990.5.1	GTTTAGGTTAGTATGAAGGATCCCAA	TGCCAGTACTCACCAATTAGTAAAAG
G1-C12	CCCAAATCCAGGAGTTCAAA	TGGGACCTGGGGCTTCATT
G1-C13	TTGCCCGAGAAAAGGAAGGACATA	CAAGGGTGGGTGAGGGACATC
G1-C5	F-CACTGTTGACTTGGGTTATGTTATT	CTGCTCCTAGGGTTTGTGAAG
G1-C7	CCCGTGTGGCTGGCTTGTGC	TCGGCTACTTTGGTGTTTTTCTCCCTCAT
G1-C9	TGGTCCGGCAACAGCATCAGA	GCTTTTCGGTATTCAACGGCAGAGTG
G1-C9	TGGTCCGGCAACAGCATCAGA	GCTTTTCGGTATTCAACGGCAGAGTG
G1-D5	AGACCCTTGCTGTGTAACTGCT	GACTGGGACTGACTTCTATGACG
G1-D6	CAGCGTGCCCACCCGATAGAA	TCCCAAAAAGTAAAATGTGCAAAGAAAA
G1-D7	CAGCGGTGGAAATGACAAACAGG	CCAAGACGACGAGGAACGGTATT
G1-E12	CAACCATGAGAGGAGGCCAGAGAAC	AAAACAGCACTACCTACAAAACCCTATT
G1-F1	TTAGTTTGGGTGCAGACAGGA	GGTGCTCAACAACAAATCAACT
G1-F20	TCCCCAACAACTCCAGCAGAT	GGAAACCACCTAGACGAAAAATG
G1-I18	CATGTTTGTGGGTGTGGTGAA	AAAAGCGGCATCTGGTAAGG
G1-I19	ACCCAGACCTGTCCACCTCA	GAACAGCTCTCCAATCCACAAG
G1-I21	CCAAGCGAAGGAGGCGTGTG	CATGTGGCTCTTTGTTTCTGGA
G1-I5	TAATCGTGTTTGGTGGCATCCTTGAGTC	AGCAGCAGTTCCATTTTCCCACACCA
G1-I8	ACCTGCAGTGGGCTAAGACC	ATGGAAATAATAAAATAAAATGTT
G1-J10	CGTTCGCTTTGCCTGCCACA	GGCTCTTCCCCGGTCGTCCAC
G1-J17	AGCGCCTTCTACACGGACAC	TATGCCCCAATTACTCTTCTGC
G1-J2	TACAGTAACTATGCCAAGATGAAATG	CAATATGGATAATGGCTGTAGACC
G1-J20	ATCCTCCAAGCTCACTACAACA	CCAGCCCCTTCCCAAACAG
G1-J9	CTGTCATTGCCTGCATCGGGGAGAAG	TGTTGAGGGGAAGCAGTTTTG
G1-K2	GCTTTCGCCTTTGACACCTC	GGCCGGACCATTGCTGAAGAAG
G1-L11	AAAGTGACCATCCAGTGCCCAAACCT	CCGGCCGAAACTGACGAGATACATTAG
G1-L13	TCAGCTGCACTAGGTTTGTC	CATTTTGATTTGCTCCATAA
G1-L19	GACAACCTTGAATCCTTTATG	AGATGTTGGTTGGTGACTTAT
G1-L20	TGGGCATAGATGGCAAGGAAAAA	CCCCCAGCATCTCGCATACAC
G1-L7	GTGCTACAGGAAGGAATGGATG	TAGCACAGGAACAGCCGACAATAA
G1-M14	CCGCTTGGACATGAGGAGAT	TGGCAAAGAAACAGAACACAACTA
G1-M19	GAGAAGTAGTGTCCCGGCAGAAAC	ATGGGTGAAAACTTAGGTGAAATG
G1-N9	GCGGGGCAATACATGACGTTCCACAG	GACCCCCATCTCCGTTTCCCATTCC
G1-O1	GGGGTAGAGCACAGTCCAGTT	TTGCAAGGCCGAAAAGGTG
G1-O12	GGAATTCCGGGGCACTACT	TCGCGAGGACGGGGAAGAG
G1-O24	CGGCCTTCCTGCAGTACAACCATC	TCGGCAACGTGAAGACCATA
G2-A11	GCCCCTGGAAGCTGTTGTGA	GGGGTCCATCCGAGTCC
G2-A7	TTACCCCACAGACAAAATCAACACC	GGCGGCCCCTCATAGCAC
G2-B1	GGGCCTAGTCCTGCTGGTC	CAAAGAGTGCGGAGAAATGG
G2-B8	CAACATGCGACCACTATAGCCACTTCCT	CGCCACCGCCACCACCACA
G2-C2	TTTGCAGGAAGAGTCATAACACAG	GTCAACAACACCCTTTTCCCTTCCT
G2-D1	GCAGGTCGGCAAGAAGCTAAAGAAGGAA	AGGGTTGGTTTGAAAGGATGTGCTGGTAA
G2-E17	GGAGCACCAAATTCAAGTCAG	CGTCCCCGGTCAATCTCCAC
G2-E19	CCAGTTTGAGCCCCAGGAG	TCGCGGCAGTCAAGAGGTC
G2-F17	TATCCTCTTATTGCTGCATTCTCCTCAC	AGTACGGCCGTTCACCATCTCTG
G2-F2	CACACCACAGACGCATTGAC	TCCCCAGCCTGTGTAGAAC
G2-G13	GGGAGGGGAGAAGGCTACCA	ATACACGGCTTCCATGCTTCTTCTT
G2-G15	CCACGGCCCCACATCCAGC	TCCCGCAGAATTTCCGTATCCAT
G2-G21	TCCAAGAGGGTGTGAGGTGAAC	AAAGCCATGCGAAGCGGAAGAC
G2-G23	GGTTTGGTACTTCAGCGGATGT	CCAAAGCCTGTACTATGCGAAAAG
G2-G5	CGGTCCCTACTGTGGTCTATGGTTTTCA	GGCTCTGCATATCCTCGGTCACACTTCC
G2-G6	CCCATGGCTGCAAGGATTACG	CAGGGGTTGTTGGGAGGCAGTGT
G2-H18	TTGTCAAATGGGCGAGTTCA	TGTTTTGCACCCAGTTTTTG
G2-I18	GATCTCCTCAGGTCTCTTTCA	GATTATGGGCCGGTGTCTCT
G2-I23	TGACTTTCCCAATGTGAGCAGAC	CAGAGGTGGTGTTACAGCAGCAGTTT
G2-J12	CCTCTTGTCCCAGTGCCAGTG	TCCAGGGATCCGAAACAAAG
G2-J21	CCGCCTCAGCCTGTTTCTCTACTTTT	CTTTGAATTTCTGCTTTTGGTGCTCTGC
G2-K12	ACATTAGTCCTGGTTACGAGAGC	AAAGGGCAGTCCAGCATTGA
G2-K2	CTGCCCAAGAAGACCGAGAGCCACAAG	AGCGCCCCCTGCACCAAAATCA
G2-L16	CCAAGGGTAGGAGAACAAGACA	ATGGCATGCTGGGAAATCA
G2-L21	GAATCTAGGTCCAAGCAGTCCCATCT	GACCATCACACCACTACCCACACTCA
G2-L3	TGAAAGAGGCCAGAAACAAGTAG	TTCCCAAGGTCTCCATAACAAT
G2-L4	TGGCCAAGAAGATGAAACAGGAAGAGGAG	TGGCAAAGGACACGACGCAGAG
G2-M14	CGGCCTCCTCGACGCATACG	CCAGGCCGGCCCATTGTTC
G2-M24	ACGGAGCACGGTCAGATTTCACG	CCCGGCTGGCTCTTCTTGCTCTT
G2-M3	CGATCCGCATTGAACGAGT	TGTGGCAGGAAGGAGAAGG
G2-N2	CGTGTTTTCCTCCTATGTCGACTTCTTTG	ACGTGCTCTGCCTTTCTTGATCTTGTGTT
G3-D7	AGGATTTCTTGGCCGGTGGAGTGG	GAAGTTGAGGGCCTGGGTGGGGAAGTA
NT001D_E08.5.1	AGAAGTTCCTAGATGAGTTGGAGGAG	AATTAATTTCCTAAACCAGGTGACAG
NT010B_E09.5.1	GAAGAGGTCCTAAAATATCAAGATGC	ATGATAGACTTCGTCCTTGTCATAGA
NT014D_E01.5.1	AAAGAAGTCCCGCATCTAACCT	ATTAAATATGAGAAGATGTGTGCAGG
V2_p1_b8	AGTCACTGTGTTACATTATCACCCAC	ATAATTATACACTGCGGTCTGCATCT
V2_p1_c5	AGTACCTGTTCGACAAGCACAC	TGAGAACATAGACAAGTTAACATACACC
V2_p1_d10	GAGATAGAAAGGCTGCATAAAGAAAT	TATGTTTCAACAATGTACAGGAAACC
V2_p1_d4	CACCAGAACAAGCTGTATTTTTATGT	TGGTTTGCATCATATATTAAAGGGTA
V2_p1_g7	GACTTCAAGCACATTGGGAAAC	ATTGTAAACTTGATAGGCTGGTGAG
V2_p2_g6	AGAATTCCCAATAGCACCTGAAAT	CACTTGGTAAATACATACACACAGCA
V2_p2_h2	CTTTTTGGCCTGGTCTTTTTG	AGATTCTTCAGACTCGTCCTTCTTT
V2_p3_a5	TTTACACAGAAACCTTGTTTATTTGGC	TTTAAGGATGCTTAGAGGCAAAGTATT
V2_p3_b1	AGTCACTGTGTTACATTATCACCCAC	TATACACTGCGGTCTGCATCTACT
V2_p5_b3	AATGGGATGAAGAGCGAGAAT	CTGCCCCATTGACATTTACCTA
V2_p5_h3	CCTTCAGACGAAAACAGCACTAAG	TACAGTGTATGAGAGCCCAATATTTC
V2_p6_a4	AGAAATACATCAAATATCGGGTGG	AAAAAGGACAATGTTCAGCTCTCT
V3_p1_a21	ACCAAGTTCTTGGAAAGTGGTG	CTTAGTGTCTCCTGGGTTTGAATAG
V3_p1_b13	GTCTTGGTACTCAATGAAGGAGATG	TCAATCTGATGAAGAGTTTACATGTCT

### Comparative gene mapping

**Figure 4:**
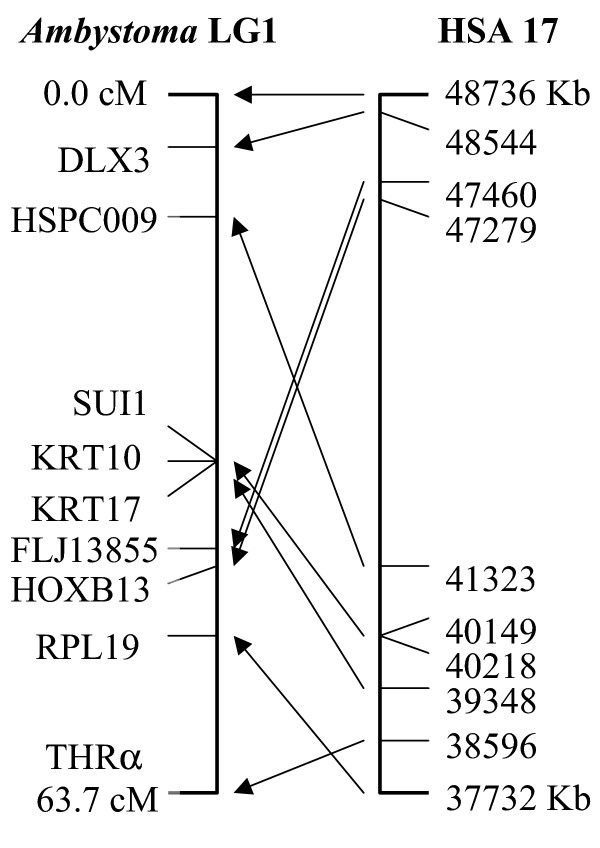
**Comparison of gene order between *Ambystoma *linkage group 1 and an 11 Mb region of Hsa17 (37.7 Mb to 48.7 Mb)**. Lines connect the positions of putatively orthologous genes.

Salamanders occupy a pivotal phylogenetic position for reconstructing the ancestral tetrapod genome structure and for providing perspective on the extremely derived anuran *Xenopus *(37) that is currently providing the bulk of amphibian genome information. Here we show the utility of ambystomatid ESTs for identifying chromosomal regions that are conserved between salamanders and other vertebrates. A region of conserved synteny that corresponds to human chromosome (*Hsa*) 17q has been identified in several non-mammalian taxa including reptiles (38) and fishes (39). In a previous study Voss et al. (40) identified a region of conserved synteny between *Ambystoma *and *Hsa *17q that included collagen type 1 alpha 1 (*Col1a1*), thyroid hormone receptor alpha (*Thra*), homeo box b13 (*Hoxb13)*, and distal-less 3 (*Dlx3) *(Figure 4). To evaluate both the technical feasibility of mapping ESTs and the likelihood that presumptive orthologues map to the same synteny group, we searched our assemblies for presumptive *Hsa *17 orthologues and then developed a subset of these loci for genetic linkage mapping. Using a joint assembly of *A. mexicanum *and *A. t. tigrinum *contigs, 97 Hsa 17 presumptive orthologues were identified. We chose 15 genes from this list and designed PCR primers to amplify a short DNA fragment containing 1 or more presumptive SNPs that were identified in the joint assembly (Table 9). All but two of these genes were mapped, indicating a high probability of mapping success using markers developed from the joint assembly of *A. mexicanum *and *A. t. tigrinum *contigs. All 6 ESTs that exhibited 'best hits' to loci within the previously defined human-*Ambystoma *synteny group did map to this region (*Hspc009*, *Sui1*, *Krt17*, *Krt24*, *Flj13855, *and *Rpl19*). Our results show that BLAST-based definitions of orthology are informative between salamanders and human. All other presumptive *Hsa *17 loci mapped to *Ambystoma *chromosomal regions outside of the previously defined synteny group. It is interesting to note that two of these loci mapped to the same ambystomatid linkage group (*Cgi-125*, *Flj20345*), but in human the presumptive orthologues are 50 Mb apart and distantly flank the syntenic loci in Figure 4. Assuming orthology has been assigned correctly for these loci, this suggests a dynamic history for some *Hsa *17 orthologues during vertebrate evolution.

**Table 9:** Presumptive human chromosome 17 loci that were mapped in *Ambystoma*

**Marker ID**	**Primers^a^**	**Diagnosis^b^**	**LG^c^**	**Symbol^d^**	**RefSeq ID^e^**	**E-value^f^**
Pl_6_E/F_6	F-GAAAACCTGCTCAGCATTAGTGT	ASA	ul	PFN1	NP_005013	E^-34^
	R-TCTATTACCATAGCATTAATTGGCAG					
Pl_5_G/H_5	F-CTATTTCATCTGAGTACCGTTGAATG	PE (A)	23	CGI-125	NP_057144	E^-56^
	R-TAATGTAGAACTAAATGGCATCCTTC					
	E-CCATGGTGCAGGAAGAGAGCCTATAT					
Pl_0.4_A/B_1	F-GTCTCATTATCCGCAAACCTGT	SP	1	RPL19	NP_000972	E^-67^
	R-ATTCTCATCCTCCTCATCCACGAC					
Pl_4_B_7/8	F-CCTAGAACATTACCAAAACAGACTCA	RD (Dpn II)	1	KRT10	NP_061889	E^-17^
	R-AATGAAGAAGTATTGCATGTGAGAAC					
Pl_4_B_9/10	F-GAACTTGTTGGCAGGTTTCTCTT	RD (AciI)	1	KRT17	NP_000413	E^-146^
	R-CTAGTGATAGGTTGGACATACCAGAG					
Pl_10_C/D_4	F-CTCCACTATTTAAAGGACATGCTACA	PE (A)	1	SUI1	NP_005792	E^-48^
	R-TTAATATAGCACAACATTGCCTCATT					
	E-TGCTACATTAATGTAATAAACGGCATCATC					
Pl_6_E/F_11	F-AAGAGAAGTTCCTAGATGAGTTGGAG	PE (A)	1	HSPC009	NP_054738	E^-26^
	R-TGAAGAGAGAACTCAAAGTGTCTGAT					
	E-TCATGTTTTGCTCTGCTGTGCAGT					
Pl_9_A/B_10	F-TGATAGTTTCTGGATTAAGACGAGTG	PE (T)	1	FLJ13855	NP_075567	E^-15^
	R-CTTAGAGCCATTGTTACAAGATGTTC					
	E-GTGATCTAGTGGGATCAAACCCTAAAGACC					
Pl_10_C/D_9	F-AAAGTGCCAAGAAGGAGATTAACTT	PE (T)	9	NME1	NP_000260	E^-71^
	R-GAGCTCAGAAAACAAGGCAGTAAC					
	E-AAATGGATCTACGAGTAGACCTTGACCC					
Pl_9_C/D_9	F-GAGTCTCCTTTAGGATTGACGTATCT	PE (T)	23	FLJ20345	NP_060247	E^-17^
	R-GCTATGTGAGCAGAGATAAAAGTCAG					
	E-GTTACAGCATCAGTGGGATGTGGTATGT					
Pl_8_C/D_9	F-AGGATACCAACCTCTGTGCTATACAT	PE (C)	15	H3F3B	NP_005315	E^-66^
	R-TAAATGTATTTACAAACCGAAAGCAA					
	E-CGTGGCGAGCGTGCCTAGT					
Pl_9_C/D_4	F-GTGGTTATTTGTAACATTTCGTTGAC	PE (A)	8	SFRS2	NP_003007	E^-40^
	R-AATTACATTTGGGCTTCTCAATTTAC					
	E-TTTTTAAACGCGTAAAAATGTTAACAGA					
Pl_6_C/D_5	F-CCGTAAATGTTTCTAAATGACAGTTG	PE (G)	2	ACTG1	NP_001605	0
	R-GGAAAGAAAGTACAATCAAGTCCTTC					
	E-GATTGAAAACTGGAACCGAAAGAAGATAAA					

^a^Sequences are 5' amplification primers, 3' amplification primers, or primer extension probes, and are preceded by F-, R-, and E- respectively. ^b^Genotyping methods are abbreviated: allele specific amplification (ASA), size polymorphism (SP), restriction digestion (RD), primer extension (PE). Diagnostic restriction enzymes and diagnostic extension bases are provided in parentheses. ^c^Ambystoma linkage group ID. "ul" designates markers that are unlinked. ^d^Official gene symbols as defined by the Human Genome Organization Gene Nomenclature Committee . ^e^Best BLASTX hit (highest e-value) from the human RefSeq database using the contig from which each marker was designed as a query sequence. ^f^Highest E-value statistic obtained by searching contigs, from which EST markers were designed, against the human RefSeq database.

### Future directions

Ambystomatid salamanders are classic model organisms that continue to inform biological research in a variety of areas. Their future importance in regenerative biology and metamorphosis will almost certainly escalate as genome resources and other molecular and cellular approaches become widely available. Among the genomic resources currently under development (see [41]) are a comparative genome map, which will allow mapping of candidate genes, QTL, and comparative anchors for cross-referencing the salamander genome to fully sequenced vertebrate models. In closing, we reiterate a second benefit to resource development in *Ambystoma*. Genome resources in *Ambystoma *can be extended to multiple, closely related species to explore the molecular basis of natural, phenotypic variation. Such extensions can better inform our understanding of ambystomatid biodiversity in nature and draw attention to the need for conserving such naturalistic systems. Several paedomorphic species, including *A. mexicanum*, are on the brink of extinction. We can think of no better investment than one that simultaneously enhances research in all areas of biology and draws attention to the conservation needs of model organisms in their natural habitats.

## Conclusions

Approximately 40,000 cDNA sequences were isolated from a variety of tissues to develop expressed sequence tags for two model salamander species (*A. mexicanum *and *A. t. tigrinum*). An approximately equivalent number of contigs were identified for each species, with 21,091 unique contigs identified overall. The strategy to sequence cDNAs from a diverse collection of tissues from normalized and non-normalized libraries yielded different sets of highly redundant contigs. Only 25% and 28% of the *A. mexicanum *and *A. t. tigrinum *contigs, respectively, were identified in common. To demonstrate the utility of these EST resources, we searched databases to identify new probes for regeneration research, characterized intra- and interspecific nucleotide polymorphism, saturated a human/*Ambystoma *synteny group with marker loci, and extended PCR primer sets designed for *A. mexicanum */ *A. t. tigrinum *orthologues to a related tiger salamander species. Over 100 new probes were identified for regeneration research using informatic approaches. With respect to comparative mapping, 13 of 15 EST markers were mapped successfully, and 6 EST markers were mapped to a previously defined synteny group in *Ambystoma*. These results indicate a high probability of mapping success using EST markers developed from the joint assembly of *A. mexicanum *and *A. t. tigrinum *contigs. Finally, we found that primer sets designed for *A. mexicanum */ *A. t. tigrinum *EST orthologues can be used to amplify the corresponding sequence in a related *A. tigrinum *complex species. Overall, the EST resources reported here will enable a diversity of new research areas using ambystomatid salamanders.

## Methods

### cDNA library construction

Ten cDNA libraries were constructed for the project using various larval tissues of *A. mexicanum *and *A. t. tigrinum *(Table 1). Larval *A. mexicanum *were obtained from adult animals whose ancestry traces back to the Axolotl Colony [17]. Larval *A. t. tigrinum *were obtained from Charles Sullivan Corp. The GARD and MATH A. mexicanum limb regeneration libraries were constructed using regenerating forelimb mesenchyme. Total RNAs were collected from anterior and posterior limbs amputated at the mid-stylopod level on 15 cm animals, and from the resulting regenerates at 12 h, 2 days, 5 days and early bud stages. One hundred μg fractions of each were pooled together and polyA-selected to yield 5 μg that was utilized for directional library construction (Lambda Zap, Stratagene). The V1 (*A. mex*), V2 (*A. tig*), V4-5 (*A. tig*), and V6-7 (*A. mex*) libraries were made from an assortment of larval tissues (see Table 1) using the SMART cDNA cloning kits (Clontech). Total RNAs were isolated and reverse transcribed to yield cDNAs that were amplified by long distance PCR and subsequently cloned into pTriplEX. The V3 and AG libraries were constructed by commercial companies (BioS&T and Agencourt, respectively).

### cDNA template preparation and sequencing

cDNA inserts were mass excised as phagemids, picked into microtitre plates, grown overnight in LB broth, and then diluted (1/20) to spike PCR reactions: (94°C for 2 min; then 30 cycles at 94°C for 45 sec, 58°C for 45°sec, and 72°C for 7 min). All successful amplifications with inserts larger than ~500 bp were sequenced (ABI Big Dye or Amersham Dye terminator chemistry and 5' universal primer). Sequencing and clean-up reactions was carried out according to manufacturers' protocols. ESTs were deposited into NCBI database under accession numbers BI817205-BI818091 and CN033008-CN045937 and CN045944-CN069430.

### EST sequence processing and assembly

The PHRED base-calling program [42] was used to generate sequence and quality scores from trace files. PHRED files were then quality clipped and vector/contaminant screened. An in-house program called QUALSCREEN was used to quality clip the ends of sequence traces. Starting at the ends of sequence traces, this program uses a 20 bp sliding window to identify a continuous run of bases that has an average PHRED quality score of 15. Mitochondrial DNA sequences were identified by searching all ESTs against the complete mtDNA genome sequence of *A. mexicanum *(AJ584639). Finally, all sequences less than 100 bp were removed. The average length of the resulting ESTs was 629 bp. The resulting high quality ESTs were clustered initially using PaCE [43] on the U.K. HP Superdome computer. Multi-sequence clusters were used as input sequence sets for assembly using CAP3 [44] with an 85% sequence similarity threshold. Clusters comprising single ESTs were assembled again using CAP3 with an 80% sequence similarity threshold to identify multi-EST contigs that were missed during the initial analysis. This procedure identified 550 additional contigs comprising 1150 ESTs.

### Functional annotation

All contigs and singletons were searched against the human RefSeq database (Oct. 2003 release) using BLASTX. The subset of sequences that yielded no BLAST hit was searched against the non-redundant protein sequence database (Feb. 2004) using BLASTX. The remaining subset of sequences that yielded no BLAST hit was searched against *Xenopus laevis *and *X. tropicalis *UNIGENE ESTs (Mar. 2004) using TBLASTX. Zebrafish ESTs were downloaded from UNIGENE ESTs (May 2004). BLAST searches were done with an E-value threshold of E <10-7 unless specified.

### Sequence comparison of *A. mexicanum *and *A. t. tigrinum *assemblies

All low quality base calls within contigs were masked using a PHRED base quality threshold of 16. To identify polymorphisms for linkage mapping, contigs from *A. mexicanum *and *A. t. tigrinum *assemblies were joined into a single assembly using CAP3 and the following criteria: an assembly threshold of 12 bp to identify initial matches, a minimum 100 bp match length, and 85% sequence identity. To identify putatively orthologous genes from *A. mexicanum *and *A. t. tigrinum *assemblies, and generate an estimate of gene sequence divergence, assemblies were compared using BLASTN with a threshold of *E *<10^-20^. Following BLAST, alignments were filtered to obtain reciprocal best BLAST hits.

### Extending *A. mexicanum */ *A. t. tigrinum *sequence information to *A. ordinarium*

Polymorphic DNA marker loci were identified by locating single nucleotide polymorphisms (SNPs) in the joint *A. mexicanum *and *A. t. tigrinum *assembly. Polymerase chain reaction (PCR) primers were designed using *Primer 3 *[45] to amplify 100 – 500 bp SNP-containing fragments from 123 different protein-coding loci (Table 8). DNA was isolated from salamander tail clips using SDS, RNAse and proteinase K treatment, followed by phenol-chloroform extraction. Fragments were amplified using 150 ng DNA, 75 ng each primer, 1.5 mM MgCl_2_, 0.25 U Taq, and a 3-step profile (94°C for 4 min; 33 cycles of 94°C for 45 s, 60°C for 45 s, 72°C for 30 s; and 72°C for 7 min). DNA fragments were purified and sequenced using ABI Big Dye or Amersham Dye terminator chemistry. Single nucleotide polymorphisms were identified by eye from sequence alignments.

### Linkage mapping of human chromosome 17 orthologous genes

Putative salamander orthologues of genes on human chromosome 17 (*Hsa *17) were identified by comparing the joint *A. mexicanum *and *A. t. tigrinum *assembly to sequences from the human RefSeq (NCBI) protein database, using BLASTX at threshold *E<*10^-7^. Linkage distance and arrangement among markers was estimated using MapManager QTXb19 software [46] and the Kosambi mapping function at a threshold of p = 0.001. All markers were mapped using DNA from a previously described meiotic mapping panel [40]. All PCR primers and primer extension probes were designed using *Primer 3 *[45] and *Array Designer2 *(Premier Biosoft) software. Species-specific polymorphisms were assayed by allele specific amplification, restriction digestion, or primer extension, using the reagent and PCR conditions described above. Primer extension markers were genotyped using the AcycloPrime-FP SNP detection assay (Perkin Elmer). See Table 9 for amplification and extension primer sequences, and information about genotyping methodology.

## Author's contributions

SP and DK: bioinformatics; JW: clone management and sequencing in support of *A. mexicanum *and *A. t. tigrinum *ESTs; JS: comparative mapping and polymorphism estimation; DW: extending ESTs to *A. ordinarium*; JM, KK, AS, NM: PCR and gel electrophoresis; BH and ET: cDNA library construction and sequencing for spinal cord regeneration ESTs; MR, SB, DG: cDNA library construction and clone management for limb regeneration ESTs; DP and SV conceived of the project and participated in its design and coordination. All authors read and approved the final manuscript.
